# Development of a novel bacterial production system for recombinant bioactive proteins completely free from endotoxin contamination

**DOI:** 10.1093/pnasnexus/pgae328

**Published:** 2024-08-07

**Authors:** Go Kamoshida, Daiki Yamaguchi, Yuki Kaya, Toshiki Yamakado, Kenta Yamashita, Moe Aoyagi, Saaya Nagai, Noriteru Yamada, Yu Kawagishi, Mizuki Sugano, Yoshiaki Sakairi, Mikako Ueno, Norihiko Takemoto, Yuji Morita, Yukihito Ishizaka, Kinnosuke Yahiro

**Affiliations:** Department of Infection Control Science, Meiji Pharmaceutical University, 2-522-1 Noshio, Kiyose, Tokyo 204-8588, Japan; Laboratory of Microbiology and Infection Control, Kyoto Pharmaceutical University, 5 Misasagi-Nakauchi-cho, Yamashina-ku, Kyoto 607-8414, Japan; Laboratory of Microbiology and Infection Control, Kyoto Pharmaceutical University, 5 Misasagi-Nakauchi-cho, Yamashina-ku, Kyoto 607-8414, Japan; Laboratory of Microbiology and Infection Control, Kyoto Pharmaceutical University, 5 Misasagi-Nakauchi-cho, Yamashina-ku, Kyoto 607-8414, Japan; Laboratory of Microbiology and Infection Control, Kyoto Pharmaceutical University, 5 Misasagi-Nakauchi-cho, Yamashina-ku, Kyoto 607-8414, Japan; Laboratory of Microbiology and Infection Control, Kyoto Pharmaceutical University, 5 Misasagi-Nakauchi-cho, Yamashina-ku, Kyoto 607-8414, Japan; Laboratory of Microbiology and Infection Control, Kyoto Pharmaceutical University, 5 Misasagi-Nakauchi-cho, Yamashina-ku, Kyoto 607-8414, Japan; Laboratory of Microbiology and Infection Control, Kyoto Pharmaceutical University, 5 Misasagi-Nakauchi-cho, Yamashina-ku, Kyoto 607-8414, Japan; Laboratory of Microbiology and Infection Control, Kyoto Pharmaceutical University, 5 Misasagi-Nakauchi-cho, Yamashina-ku, Kyoto 607-8414, Japan; Laboratory of Microbiology and Infection Control, Kyoto Pharmaceutical University, 5 Misasagi-Nakauchi-cho, Yamashina-ku, Kyoto 607-8414, Japan; Department of Infection Control Science, Meiji Pharmaceutical University, 2-522-1 Noshio, Kiyose, Tokyo 204-8588, Japan; Department of Infection Control Science, Meiji Pharmaceutical University, 2-522-1 Noshio, Kiyose, Tokyo 204-8588, Japan; Department of Intractable Diseases, National Center for Global Health and Medicine, 1-21-1 Toyama, Shinjyuku-ku, Tokyo 162-8655, Japan; Pathogenic Microbe Laboratory, Research Institute, National Center for Global Health and Medicine, 1-21-1 Toyama, Shinjyuku-ku, Tokyo 162-8655, Japan; Department of Infection Control Science, Meiji Pharmaceutical University, 2-522-1 Noshio, Kiyose, Tokyo 204-8588, Japan; Department of Intractable Diseases, National Center for Global Health and Medicine, 1-21-1 Toyama, Shinjyuku-ku, Tokyo 162-8655, Japan; Laboratory of Microbiology and Infection Control, Kyoto Pharmaceutical University, 5 Misasagi-Nakauchi-cho, Yamashina-ku, Kyoto 607-8414, Japan

**Keywords:** *Acinetobacter baumannii*, endotoxin, recombinant protein expression, VHH

## Abstract

Endotoxins, or lipopolysaccharides (LPS), are potent immunostimulatory molecules of critical concern in bacterial recombinant protein expression systems. The gram-negative bacterium *Acinetobacter baumannii* exhibits an interesting and unique phenotype characterized by the complete loss of LPS. In this study, we developed a novel system for producing recombinant proteins completely devoid of endotoxin contamination using LPS-deficient *A. baumannii*. We purified endotoxin-free functional green fluorescent protein, which reduced endotoxin contamination by approximately three orders of magnitude, and also purified the functional cytokine tumor necrosis factor (TNF)-α. Additionally, utilization of the Omp38 signal peptide of *A. baumannii* enabled the extracellular production of variable domain of heavy chain of heavy chain (VHH) antibodies. With these advantages, mNb6-tri-20aa, a multivalent VHH that specifically binds to the spike protein of severe acute respiratory syndrome coronavirus 2, was purified from the culture supernatant, and endotoxin contamination was reduced by a factor of approximately 2 × 10^5^ compared with that in conventional expression systems. A virus neutralization assay demonstrated the functionality of the purified antibody in suppressing viral infections. Moreover, we applied our system to produce ozoralizumab, a multispecific VHH that binds to human TNF-α and albumin and are marketed as a rheumatoid arthritis drug. We successfully purified a functional antibody from endotoxin contamination. This system establishes a new, completely endotoxin-free platform for the expression of recombinant proteins, which distinguishes it from other bacterial expression systems, and holds promise for future applications.

**Significance Statement**

Bacterial recombinant protein expression systems are essential tools in modern science and biopharmaceutical production. Endotoxins pose a critical concern as potent immunostimulatory molecules in these systems. To date, no entirely endotoxin-free recombinant protein expression system has been developed using bacteria. *Acinetobacter baumannii* exhibits an interesting and unique phenotype as it shows no endotoxin activity owing to the complete loss of lipopolysaccharides. We developed a novel system for producing recombinant proteins using this bacterium, and successfully purified bioactive proteins free of endotoxin contamination. Our protein expression system represents the first completely endotoxin-free platform, which distinguishes it from other bacterial expression systems and is expected to be applied in various fields, including clinical practice, in the future.

## Introduction

*Escherichia coli*, a gram-negative bacterium, has been utilized as a model organism since the dawn of molecular biology and has been established as a host for protein production. Many tools available for protein expression in *E. coli* perform significantly better than those used in other bacterial or cell culture systems (1, 2). Nonetheless, the presence of lipopolysaccharides (LPS), powerful immunostimulatory endotoxins in the outer membrane of gram-negative bacteria, is crucial. In mammals, endotoxins can cause febrile reactions and ultimately septic shock (3). Consequently, to ensure the safe administration of recombinant biopharmaceuticals expressed in *E. coli* in humans, it is imperative to eliminate any contaminating LPS. To date, no method has been devised to eliminate endotoxins (4–6). The *Limulus* amebocyte lysate (LAL) assay, the gold standard for detecting endotoxin contamination, utilizes aqueous extracts of *Limulus polyphemus* blood cells and is the most sensitive and reliable method for the in vitro detection of bacterial endotoxins (7, 8). The LAL assay was approved by the Food and Drug Administration in the 1970s, and the Pharmacopeial Discussion Group has designated it as the exclusive endotoxin testing method in Japan, the USA, and Europe.

Recently, biopharmaceuticals such as small-molecule antibodies, including the variable domain of heavy chain of heavy chain (VHH) and scFv, have garnered attention as novel modalities for drug discovery (9–11). Although they can be expressed in bacteria, they frequently form inclusion bodies (12–14). Many biopharmaceuticals are currently produced using cultured mammalian and insect cells, which results in significantly high production costs (15). Bacterial expression systems are inapplicable to certain products because of molecular weight limitations and the absence of glycosylation; however, their production costs are significantly lower than those of cultured cell systems (16). Camelids possess antibodies consisting solely of heavy chains. This binding domain is frequently referred to as VHH or nanobody. VHH are amenable to bacterial production because of their small size (∼15 kDa) and lack of glycosylation sites. Compared with conventional antibodies, VHH antibodies are characterized by greater stability and the ability to retain activity even under adverse conditions such as fluctuating temperature and pH (17, 18). Additionally, they can be readily modified through protein engineering techniques, such as the creation of multivalent antibodies, multispecific antibodies, and antibody-drug conjugates. Therefore, VHH antibodies have great potential for commercial applications in medical and diagnostic fields (11, 19–21).

Although *Acinetobacter baumannii* is renowned for its proclivity toward drug resistance and nosocomial infections, certain strains that are resistant to LPS-targeting agents (such as colistin) exhibit an intriguing phenotype characterized by the complete loss of LPS and diminished virulence (22, 23). LPS is typically deemed an essential component for the survival of gram-negative bacteria; however, *A. baumannii* has the potential to completely lose LPS through mutations in the *lpxACD* genes responsible for LPS biosynthesis (24–26). This study aimed to develop an innovative bacterial system for endotoxin-free production of recombinant proteins using a strain completely lacking LPS.

## Results and discussion

### Potential of endotoxin-free strains as hosts for recombinant protein expression

We recently reported that the colistin-resistant *A. baumannii* KL037S strain, established by colistin selection, exhibits notably diminished endotoxin levels (24). These findings prompted the development of an endotoxin-free protein expression system using an LPS-deficient *A. baumannii* strain. The endotoxin concentration of the bacterial resuspension of colistin-resistant *A. baumannii* KL037S strain, at an optical density at 600 nm (OD_600_) of 0.1, was 0.030 ± 0.002 EU/mL, whereas that of the parental wild-type strain (ATCC 19606) was 3.3 × 10^4^ ± 1.0 × 10^4^ EU/mL (Table 1). Endotoxin concentrations in drinking water have been reported to be 3–15 EU/mL (27, 28). Additionally, the endotoxin content in water for injection is <0.25 EU/mL (in Japan and Europe) and <0.5 EU/mL (in the USA) according to the pharmacopeia of each country. The endotoxin level in the KL037S resuspension (OD_600_ of 0.1) was approximately 1/100th of that in drinking water, making it sufficiently low for comparison with water for injection.

**Table 1.** Endotoxin levels of each bacterial species.

Bacteria	ATCC 19606	KL037S	ClearColi
Endotoxin level (EU/mL OD_600_ of 0.1)	3.3 × 10^4^ ± 1.0 × 10^4^	0.030 ± 0.002	2.1 × 10^3^ ± 0.033 × 10^3^

**Fig. 1.**
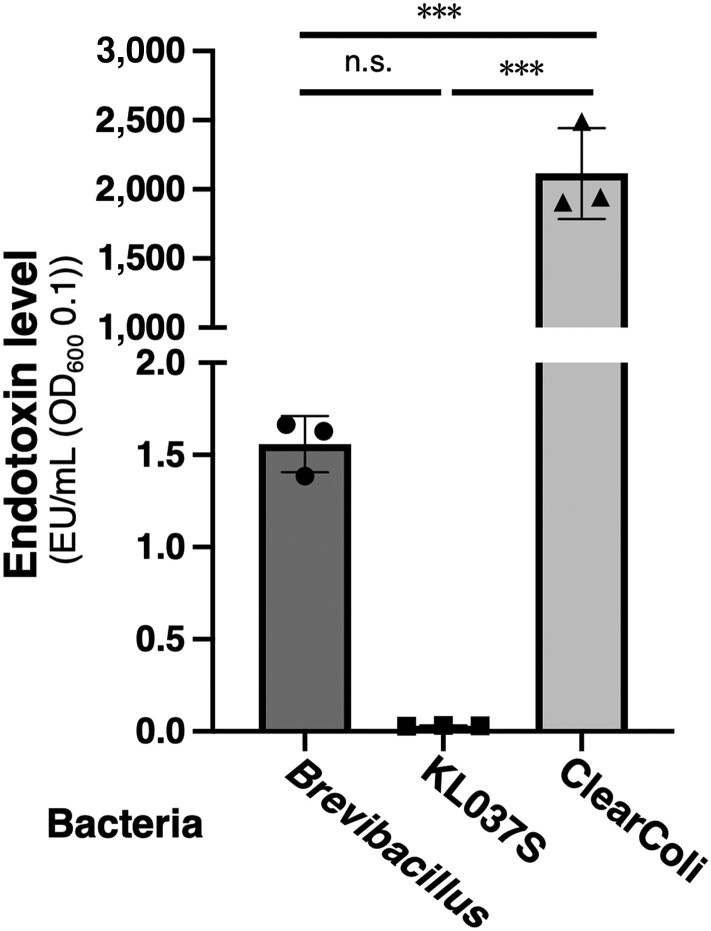
Endotoxin levels of each bacterial species. *Brevibacillus*, KL037S, and ClearColi cell resuspensions were adjusted to an OD_600_ of 0.1, and endotoxin levels were measured using the *Limulus* amebocyte lysate assay. Data were obtained from three biological replicates. Mean values and error bars represent the standard deviation. ***P < 0.001, n.s., not significant.

To reduce endotoxin contamination, ClearColi was engineered through genetic manipulation of the *E. coli* BL21(DE3) strain, resulting in conversion of the hexaacyl lipid A of LPS to tetraacyl lipid A (lipid IV_A_). Unlike typical bacterial LPS, the lipid IV_A_ does not induce an endotoxic response in humans (13, 29–31). However, coagulation of LAL is induced in the presence of lipid IV_A_, yielding a positive result in the LAL assay (30). In this study, the endotoxin concentration of ClearColi was 2.1 × 10^3^ ± 0.33 × 10^3^ EU/mL, which was approximately 70,000-fold higher than that in the KL037S strain (Table 1 and Fig. 1). Recombinant proteins produced using ClearColi can substantially decrease endotoxin levels, although residual amounts remain detectable (30, 31). Recently, a protein expression system was developed using *Sphingobium* sp., a gram-negative bacterium that naturally possesses sphingoglycolipids instead of LPS. However, the yield remained low (80 µg/L); hence, further improvement is required (32). Furthermore, *Sphingobium* sp. yielded positive results in the LAL assay (33). We also observed that in *Brevibacillus*, a gram-positive bacterium used as a host for protein expression, the LAL assay signal was 1.6 ± 0.15 EU/mL, which is approximately 53-fold higher than that in the KL037S strain (Fig. 1). LAL coagulation is also induced by fungal β-glucans (7, 8) and lipoteichoic acid in gram-positive bacteria (34, 35). Thus, the LAL assay has never yielded negative results for microorganisms. Therefore, we propose the use of the KL037S strain as a host for endotoxin-free recombinant protein production for pharmaceuticals.

**Fig. 2.**
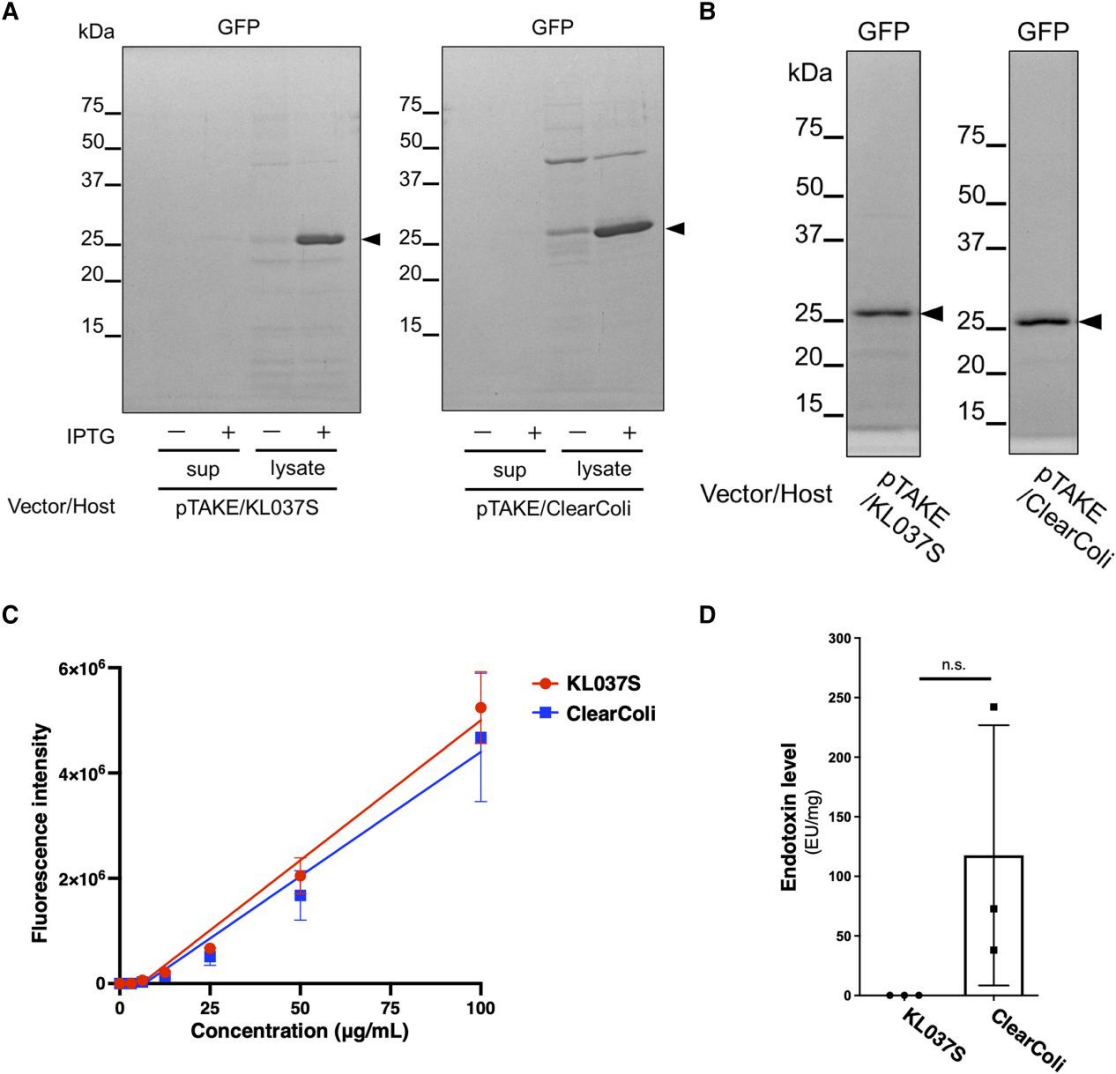
Evaluation of GFP purified from endotoxin-reduced cell systems. A) GFP fractions purified from KL037S and ClearColi with pTAKE-GFP were observed using sodium dodecyl sulfate-polyacrylamide gel electrophoresis. The culture supernatants (sup) and cell lysates (lysate) from the 2-mL culture with or without isopropyl-β-D-thiogalactopyranoside (IPTG) induction were analyzed. Whole proteins were visualized using CBB staining. B) Purified GFP fractions from 100-mL KL037S and ClearColi lysates after IPTG induction were analyzed. Whole proteins were visualized using CBB staining. Arrowheads indicate GFP. C) Purified GFP from the indicated strains was diluted to 10 µg/100 µL, and a 2-fold dilution series with saline was used to observe fluorescence signals. Data are expressed as the means from three independently purified samples, and error bars represent the standard deviation. D) Endotoxin levels in purified GFP fractions from the indicated strains were measured using the *Limulus* amebocyte lysate assay. Protein levels were quantified using a BCA protein assay. Data are expressed as the means from three independently purified samples, and error bars represent the standard deviation. n.s., nonsignificant.

First, green fluorescent protein (GFP), a widely accepted gold standard for recombinant proteins, was produced in KL037S. We developed pTAKE, an *E. coli-Acinetobacter* shuttle vector for recombinant protein expression (Supplementary Fig. S1). pTAKE contains a *tac*-promoter followed by a *lac*-operator sequence, and expression of the downstream gene can be induced in the presence of isopropyl-β-D-thiogalactopyranoside (IPTG). The plasmid was designed to express recombinant proteins with a His-tag and an FXa cleavage site at the N-terminus. After subcloning the GFP gene into pTAKE and introducing the resultant plasmid into KL037S or ClearColi, we purified His-tagged GFP on a 2-mL scale from the cell lysate using Ni-NTA agarose and compared the purified fractions using Coomassie brilliant blue (CBB) staining (Fig. 2A). The number of other contaminant proteins in the purified GFP fraction was lower in KL037S than that in ClearColi, indicating the high potential of both pTAKE and KL037S as protein expression systems. Scaling up to a 100-mL batch culture showed no adverse effects (Fig. 2B).

We determined whether GFP purified from KL037S functioned using a dilution series, and observed a fluorescence activity level comparable to that of GFP purified from ClearColi (Fig. 2C and Supplementary Fig. S2). The yield of GFP was 0.38 ± 0.16 mg/100 mL for KL037S and 1.7 ± 0.25 mg/100 mL for ClearColi, with KL037S yielding approximately one quarter that of ClearColi (Table 2). In contrast, endotoxin levels in purified GFP fractions were 0.14 ± 0.01 EU/mg for KL037S and 117 ± 109 EU/mg for ClearColi, demonstrating a considerable reduction of approximately 1/836th in KL037S compared with that in ClearColi (Fig. 2D and Table 2). Thus, the recombinant protein purified from endotoxin-free KL037S exhibited remarkably low endotoxin levels.

**Table 2.** Endotoxin levels and yields of GFP produced by the endotoxin-free and ClearColi systems.

Protein	GFP	GFP
Vector	pTAKE	pTAKE
Host	KL037S Lysate	ClearColi Lysate
Endotoxin level (EU/mg)	0.14 ± 0.01	117 ± 109
Yield (mg/100 mL)	0.38 ± 0.16	1.7 ± 0.25

### Production of mouse tumor necrosis factor-α using the endotoxin-free strain

**Fig. 3.**
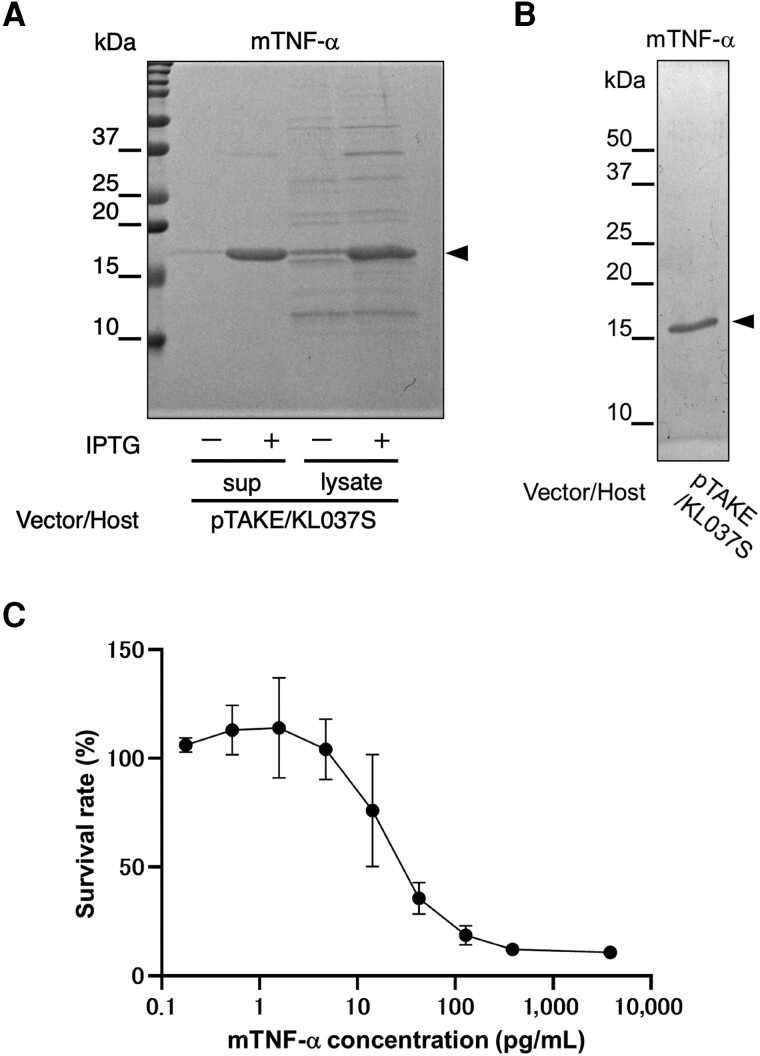
Expression and purification of mouse tumor necrosis factor (mTNF)-α using the endotoxin-free system and functional evaluation. A) mTNF-α fractions purified from the culture supernatants (sup) and cell lysates (lysate) from the 2-mL culture with or without isopropyl-β-D-thiogalactopyranoside (IPTG) induction of KL037S with pTAKE-mTNF-α were analyzed. Whole proteins were visualized using CBB staining. B) Purified mTNF-α fractions from the supernatant of the 100-mL KL037S culture with IPTG induction. The fractions were separated by electrophoresis and visualized by CBB staining. Arrowheads indicate mTNF-α. C) Dose-response curves of mTNF-α activity in the L929 cell death assay. mTNF-α was serially diluted 3-fold from 3800 pg/mL. Survival rates were calculated as the percentage of untreated cells. Data are expressed as the mean ± SD from three independently purified samples.

Next, we aimed to produce the cytokine mouse tumor necrosis factor (mTNF)-α. The mouse-derived *tnfa* gene was inserted into the cloning sites of pTAKE, expressed in KL037S, and the mTNF-α was purified (Fig. 3A). Notably, mTNF-α was also secreted into the culture supernatant, resulting in a higher degree of purity and lower levels of other contaminant proteins than in the cell lysate. When we purified mTNF-α from the culture supernatant of 100 mL of batch culture, the yield and endotoxin levels were 0.12 ± 0.04 mg/100 mL and 3.6 ± 2.0 EU/mg, respectively (Fig. 3B and Table 3).

**Table 3.** Endotoxin levels and yields of tumor necrosis factor (TNF)-α produced by the endotoxin-free system.

Protein	mTNF-α
Vector	pTAKE
Host	KL037S Supernatant
Endotoxin level (EU/mg)	3.6 ± 2.0
Yield (mg/100 mL)	0.12 ± 0.04

Furthermore, we assessed the activity of purified mTNF-α using a cell viability assay with TNF-sensitive mouse fibroblasts. Cell viability decreased in a concentration-dependent manner, with an effective half-maximal concentration (EC_50_) of 24 pg/mL (Fig. 3C). Endotoxin levels of commercial recombinant mTNF-α products are typically listed at ∼<100–1,000 EU/mg. Compared with the current commercial recombinant protein products, our endotoxin-free protein expression platform could purify mTNF-α with extremely low endotoxin levels. We also attempted to produce other cytokines. Mouse interferon (mIFN)-γ could be purified from the cell lysate fraction, but mouse granulocyte-colony stimulating factor (mG-CSF) could not be purified using pTAKE and KL037S (Supplementary Fig. S3). Future efforts to produce diverse cytokines will demonstrate the effectiveness of our proposed system.

### Production of VHH antibodies using an endotoxin-free recombinant protein expression platform

**Fig. 4.**
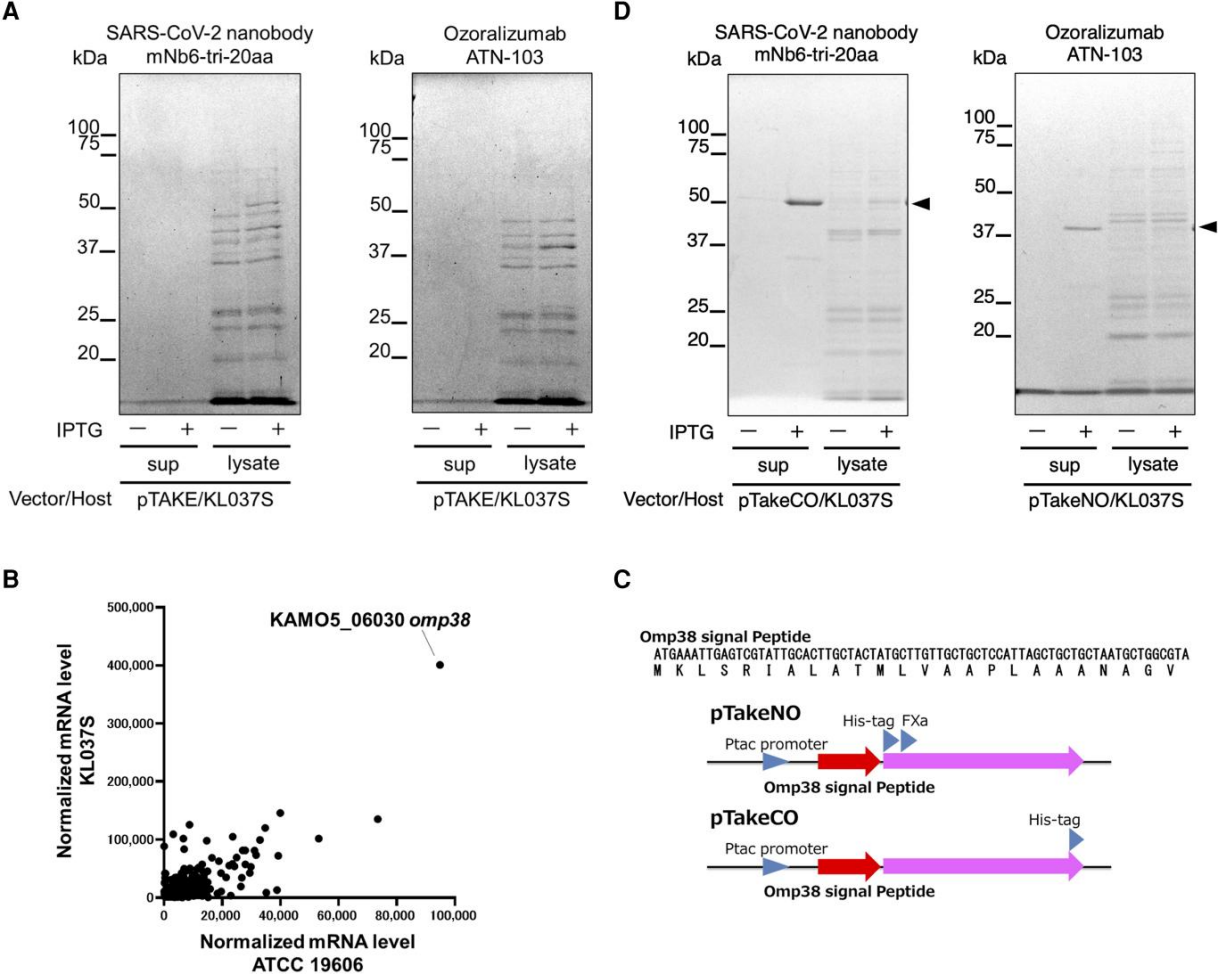
Expression and purification of antibodies using the endotoxin-free system. mNb6-tri-20aa (SARS-CoV-2 nanobody) and ozoralizumab (ATN-103) were purified from the culture supernatants (sup) and cell lysates (lysate) from the 2-mL culture with or without isopropyl-β-D-thiogalactopyranoside induction of KL037S. The experiments were conducted using the KL037S strain harboring pTAKE-mNb6-tri-20aa or pTAKE-ozoralizumab A) and pTakeCO-mNb6-tri-20aa or pTakeNO-ozoralizumab D). The purified antibody fractions were separated by electrophoresis and detected by CBB staining. Arrowheads indicate mNb6-tri-20aa (left panel) and ozoralizumab (right panel). B) mRNA expression levels in the ATCC 19606 and KL037S strains were analyzed by RNA sequencing and are represented as a scatter plot. The vertical and horizontal axes represent the normalized mRNA expression levels in the KL037S and ATCC 19606 strains, respectively. C) The nucleotide and translated amino acid sequence of the Omp38 signal sequence and a schematic diagram of the pTakeNO/CO vectors are shown. Both pTakeNO/CO vectors have a *tac*-promoter and Omp38 signal sequence. pTakeNO expresses proteins with a His-tag and an FXa site at the N-terminus, whereas pTakeCO expresses proteins with a His-tag at the C-terminus.

Recent advances in the field of VHH antibodies have encouraged the development of this endotoxin-free platform. To demonstrate its utility, we generated two VHH antibodies. One candidate was mNb6-tri-20aa, which was engineered to exhibit improved virus-neutralizing activity by linking three VHHs that specifically bind to the spike protein of severe acute respiratory syndrome coronavirus 2 (SARS-CoV-2), a multivalent antibody (36). Another candidate was the multispecific antibody ozoralizumab (ATN-103), which combines two antihuman TNF-α VHHs and one antihuman albumin VHH and has been approved as a rheumatoid arthritis drug in Japan (37–39). Despite using pTAKE and KL037S, we could not produce either of the candidate antibodies, mNb6-tri-20aa or ozoralizumab, from either the culture supernatant or cell lysate fraction (Fig. 4A).

Some proteins, such as antibodies, may be difficult to express in bacteria owing to their solubility. A previous study demonstrated that mNb6-tri-20aa was successfully expressed in a pET26b(+) plasmid in *E. coli* (BL21(DE3)) (36). Similarly, we expressed mNb6-tri-20aa using a pET26(+) plasmid in *E. coli* (BL21(DE3)) (Supplementary Fig. S4). The pET26b(+) vector harbors the *pelB* signal sequence and was designed to add a periplasmic transfer signal peptide to the N-terminus of the target protein to improve the solubility of the target protein. To identify the periplasmic transfer signal sequences compatible with LPS-deficient *A. baumannii*, we performed RNA sequencing analysis of KL037S and its parent strain ATCC 19606. The scatter plot of mRNA expression levels in KL037S and ATCC 19606 revealed that *omp38* (KAMO5_06030), which encodes an outer membrane protein and contains a putative periplasmic transfer signal, was the highest in both strains (Fig. 4B). Therefore, we constructed plasmid pTakeNO/CO vectors containing the Omp38 signal peptide at the N-terminus of recombinant proteins (Fig. 4C). The pTakeCO vector expresses proteins with a His-tag at the C-terminus, whereas the pTakeNO vector expresses proteins with a His-tag and an FXa site incorporated at the N-terminus. Surprisingly, the combination of KL037S with pTakeCO-mNb6-tri-20aa or pTakeNO-ozoralizumab produced the SARS-CoV-2 nanobody mNb6-tri-20aa or ozoralizumab (ATN-103), respectively, in the culture supernatants (Fig. 4D). VHH antibodies were also expressed in the culture supernatants when the His-tag position was changed using the pTakeNO/CO vector (Supplementary Fig. S5). Therefore, implementation of the Omp38 signal peptide enabled efficient extracellular secretory production of VHH antibodies. Purification of recombinant proteins from culture supernatants is convenient, and cell lysis or other complex procedures are not necessary, which minimizes endotoxin contamination.

We also tested the effect of the Omp38 signal peptide on mTNF-α expression, which was purified from both the culture supernatant and cell lysate fractions using pTAKE. Unfortunately, when pTakeNO was used, mTNF-α expression decreased slightly in both fractions, and when pTakeCO was used, it was not expressed (Supplementary Fig. S6). These results suggest that the effect of the Omp38 signal peptide depends on the target protein and may be particularly favorable for small-molecule antibodies. Although further research is required to fully realize this potential, we demonstrated that using the Omp38 signal peptide enabled the extracellular secretory production of two endotoxin-free antibodies.

**Fig. 5.**
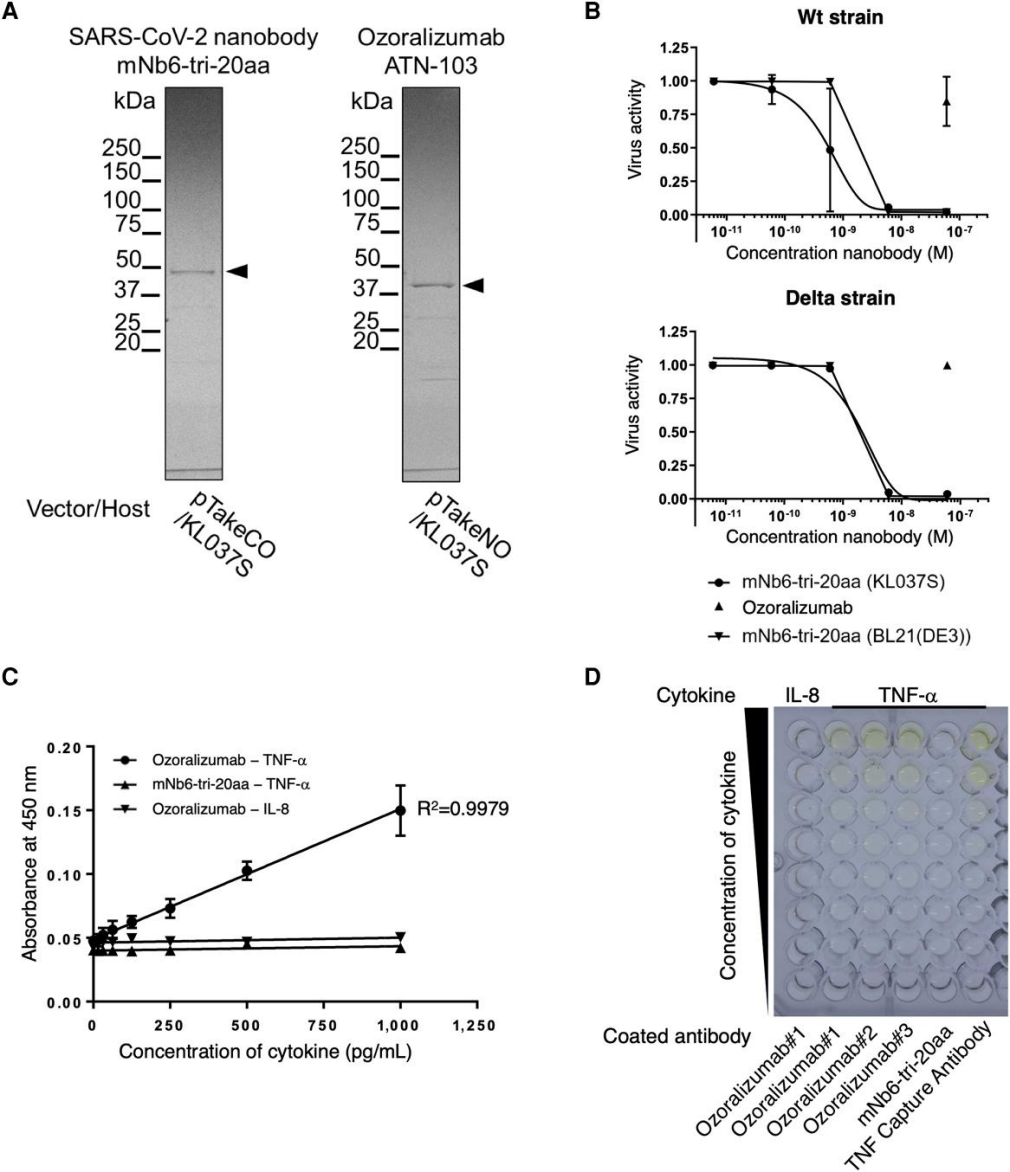
Functional evaluation of antibodies: mNb6-tri-20aa against SARS-CoV-2 and ozoralizumab against human tumor necrosis factor (TNF)-α. A) mNb6-tri-20aa (SARS-CoV-2 nanobody) and ozoralizumab (ATN-103) fractions purified from the culture supernatants of 100-mL KL037S with isopropyl-β-D-thiogalactopyranoside were analyzed. The purified antibody fractions were isolated by electrophoresis and detected by CBB staining. Arrowheads indicate antibodies. B) The neutralizing activity of antibodies against SARS-CoV-2 was evaluated using a virus neutralization assay in VeroE6 cells. The upper panel represents the wild-type (Wuhan) strain, and the lower panel represents the delta strain of SARS-CoV-2. The mNb6-tri-20aa concentration ranged from 6 × 10^−8^ M to a 10-fold dilution series. The circles represent mNb6-tri-20aa purified from KL037S culture supernatants. Data are expressed as the mean ± SD from three independently purified samples. The up-triangle represents ozoralizumab at a concentration of 6 × 10^−8^ M. Data are expressed as the mean ± SD from two independently purified samples. The down-triangle represents mNb6-tri-20aa purified from the BL21(DE3) lysate (*n* = 1 purified sample). Viral activity was calculated as a percentage of the untreated cells. ELISA was performed using ozoralizumab as the capture antibody (coated concentration of 4 µg/mL) to evaluate whether ozoralizumab recognizes human TNF-α. Quantitated dose-dependence C) and photographs of an analyzed 96-well plate D). The added human TNF-α or interleukin (IL)-8 concentrations ranged from 1,000 pg/mL to a 10-fold dilution series. Data are expressed as the mean ± SD from three independently purified samples.

Finally, two antibodies, mNb6-tri-20aa and ozoralizumab, were purified from KL037S culture supernatants, and their endotoxin levels and functionality were evaluated (Fig. 5A). The yield and endotoxin level of mNb6-tri-20aa were 0.13 ± 0.01 mg/100-mL culture and 0.26 ± 0.06 EU/mg, respectively, whereas those of ozoralizumab were 0.11 ± 0.02 mg/100 mL and 0.99 ± 0.50 EU/mg, respectively (Table 4). Endotoxin tolerances for pharmaceuticals are established by the United States Pharmacopeia and other authorities at 0.2 EU/kg for intrathecal injection drugs and 5 EU/kg for other products administered parenterally (40). Ozoralizumab was administered subcutaneously at a dose of 30 mg, which amounts to approximately 30 EU and complies with the criteria, despite being crudely purified in this study.

**Table 4.** Endotoxin levels and yields of variable domain of the heavy chain antibodies produced by the endotoxin-free and *Escherichia coli* systems.

Protein	mNb6-tri-20aa	mNb6-tri-20aa	ATN103-FL
Vector	pET26b(+)	pTakeCO	pTakeNO
Host	BL21(DE3) Lysate	KL037S Supernatant	KL037S Supernatant
Endotoxin level (EU/mg)	5.9 × 10^4^ ± 4.6 × 10^4^	0.26 ± 0.06	0.99 ± 0.50
Yield (mg/100 mL)	0.22 ± 0.07	0.13 ± 0.01	0.11 ± 0.02

The activity of the SARS-CoV-2 nanobody mNb6-tri-20aa was evaluated using a coronavirus neutralization assay (41). Preincubation of the virus with purified mNb6-tri-20aa successfully increased the viability of cells in an antibody concentration-dependent manner, revealing that viral activity was neutralized for both the wild-type (Wuhan) and delta-variant strains of SARS-CoV-2 (Fig. 5B). As expected, ozoralizumab did not exhibit neutralizing activity against SARS-CoV-2, even at high concentrations. The half-maximal inhibitory concentrations (IC_50_) of mNb6-tri-20aa derived from KL037S were 820 pM and 1.9 nM for the wild-type and delta-variant SARS-CoV-2 strains, respectively. Similarly, the IC_50_ for mNb6-tri-20aa purified from BL21(DE3) lysate was 1.9 nM for both strains. The IC_50_ of mNb6-tri-20aa was previously reported to be 160 pM for the wild-type SARS-CoV-2 (36). The findings also showed that mNb6-tri-20aa exhibited neutralizing activity against the delta-variant strains.

The activity of a multispecific VHH ozoralizumab, which binds to human TNF-α, was evaluated using enzyme-linked immunosorbent assay (ELISA). The capture antibody in the human TNF-α ELISA kit was replaced with ozoralizumab. The results showed a TNF-α concentration-dependent increase in the ELISA signal, with a correlation coefficient of *R*² = 0.9979 (Fig. 5C). Conversely, the negative control cytokine interleukin (IL)-8 was not detected. Moreover, when the negative control antibody mNb6-tri-20aa was used as the capture antibody, no TNF-α-dependent signal was observed (Fig. 5D). These results indicated that the two antibodies expressed on our platform were functional.

We further attempted to purify the two antibodies, mNb6-tri-20aa and ozoralizumab, using a combination of ClearColi and pET26b(+). The results confirmed the expected bands on the 2-mL scale (Supplementary Fig. S7A). When scaled up to 100 mL, the target protein could not be purified because of the lack of bacterial growth, which was possibly caused by toxicity. Therefore, mNb6-tri-20aa was purified from cell lysates using the *E. coli* strain BL21(DE3) and pET26b(+). Although we attempted a 100-mL scale purification of mNb6-tri-20aa with this combination, numerous contaminant proteins and high endotoxin levels (5.9 × 10^4^ ± 4.6 × 10^4^ EU/mg) were detected (Table 4 and Supplementary Fig. S7B).

### Challenges and opportunities for advancement within the novel recombinant protein expression system

LPS deficiency in endotoxin-free strains, such as KL037S, incurs significant fitness costs. KL037S showed notably diminished growth potential compared with that of the parental strain ATCC 19606 (Supplementary Fig. S8) (24). We believe that this reduced proliferative capacity is because of the instability of the outer membrane of the LPS-deficient strain. If this issue can be addressed, this may lead to an increase in protein yield. The suboptimal growth potential of the host strain indicates the need for improvement. As mentioned above, there is ample scope for enhancing the yield of recombinant proteins, but it is currently lower than that of existing *E. coli* expression systems. In this study, the yield of GFP purified using ClearColi was 1.7 mg/100 mL, whereas the yield of GFP purified using this system was 0.38 mg/100 mL (Table 2). The purified fractions from the cell lysate and culture supernatant were different, and the purity varied. The yield was 0.22 mg/100 mL for the BL21(DE3)-derived mNb6-tri-20aa (SARS-CoV-2 nanobody), whereas it was 0.13 mg/mL for this system (Table 4). An automated inducible expression system, as previously employed using ClearColi (42), could improve the yields with this endotoxin-free strain. A limitation of this study lies in the use of *A. baumannii* as a host for protein expression. Future studies should focus on understanding the mechanism by which *A. baumannii* achieves LPS deficiency and replicating this in *E. coli*, because the array of tools available for protein expression in this system far surpasses those in alternative systems.

Our novel system constitutes a completely endotoxin-free platform for recombinant protein expression, which has not yet been achieved in any other microorganism-based expression system. Endotoxin contamination is a significant concern in basic research and is likely to have detrimental effects. At present, when investigating the functions and biological effects of target proteins expressed and purified in *E. coli*, minimal consideration is given to LPS contamination (43). To accurately evaluate the functions and effects of target proteins and discern realistic reactions, it is imperative to establish a protein preparation method that is completely free of LPS contamination. This system serves as a highly valuable tool and has immense potential for application in fields, such as regenerative medicine and cell differentiation research, where even minimal endotoxin contamination can pose problems.

This system is noteworthy because it enables the secretion of recombinant proteins such as VHH antibodies into the culture supernatant. Using this system, we produced VHH antibodies (mNb6-tri-20aa and ozoralizumab) that are usually difficult to express in *E. coli*. We enabled the expression of recombinant proteins with endotoxin-free functionality, which is challenging for existing expression systems. Ozoralizumab is produced using Chinese hamster ovarian cells as a host organism (Japanese Pharmaceutical Interview Form: 873999), making production costs a formidable challenge. This study demonstrates the capability of an economical bacterial system to produce endotoxin-free and functional ozoralizumab. The secretion ability of the expression system, although requiring large-scale handling, is relatively simple and thus amenable to automation using a perfusion culture, with the potential for industrial applications.

## Materials and methods

### Bacterial strains and isolation of endotoxin-free strains

BL21(DE3), an *E. coli* strain for protein expression, and ClearColi, an *E. coli* strain with a reduced endotoxin response, were purchased from New England BioLabs (Hitchin, UK) and Lucigen (Middleton, WI, USA), respectively. For gram-positive bacterial protein expression, *B. brevis* was purchased from Takara Bio Inc. (Shiga, Japan). *A. baumannii* ATCC 19606 was obtained from the ATCC (Manassas, VA, USA).

KL037S, an endotoxin-free strain, was previously isolated (24). Briefly, ATCC 19606 was directly plated onto Luria–Bertani (LB) agar (BD Biosciences, San Diego, CA, USA) containing 10 µg/mL colistin sulfate (FUJIFILM Wako Pure Chemical Industries, Osaka, Japan) and incubated for 24 h at 37°C. Among the colonies that formed on the colistin-containing plate, an LPS-deficient strain with a deletion mutation of 215 bp in the *lpxC* gene was obtained.

### LAL assay

The LAL assay was performed using a Toxicolor LS-50M kit (Seikagaku Corporation, Tokyo, Japan), according to the manufacturer's protocol. To quantify the endotoxins in the bacteria, overnight bacterial cultures were washed with pyrogen-free saline (Otsuka Pharmaceutical Co., Ltd., Tokyo, Japan) and adjusted to an OD_600_ of 0.1; if necessary, the cultures were diluted with pyrogen-free saline and measured. The quantity of endotoxins in the protein samples was calculated in EU/mg, based on the amount of protein quantified using a bicinchoninic acid (BCA) protein assay kit (Thermo Fisher Scientific, Waltham, MA, USA).

### RNA sequencing analysis

Bacterial cultures were grown in LB broth at 37°C with constant shaking until the OD_600_ reached 0.7. Total RNA was extracted from 1 × 10^9^ colony-forming units of cells using NucleoSpin RNA (Takara Bio Inc.), according to the manufacturer's protocol. Additional treatment with DNase (Takara Bio Inc.) was performed to completely remove the genomic DNA. The removal of rRNA (NEBNext rRNA Depletion Kit, New England BioLabs), preparation of RNA libraries using the MGIEasy RNA Directional Library Prep Set (MGI Tech Co., Ltd., Wuhan, China), and acquisition of 2 × 150 bp sequences using a DNBSEQ-G400RS sequencer (MGI Tech Co., Ltd.) were outsourced to Genome-Lead (Kagawa, Japan). The sequencing data were registered with the DNA Data Bank of Japan (DDBJ) (BioProject accession no. PRJDB16719). Sequencing data were trimmed using Trim-Galore v0.6.10 and mapped to ATCC 19606 (nucleotide accession no. AP025740 in DDBJ) using HISAT2 v2.2.1 (44) to obtain count data. Subsequently, count data were normalized using the DESeq2 v1.34.0 package (Bioconductor) (45).

### Preparation of expression plasmids

The plasmid pET26b(+), a protein expression vector in *E. coli*, was purchased from Merck Millipore (Burlington, MA, USA). The plasmid pTAKE, an *E. coli-Acinetobacter* shuttle vector for recombinant protein expression, was constructed as follows: the IPTG-inducible protein expression cassette comprising the *lacI*-*tac*-promoter region of pMAL-c2e (New England Biolabs Inc., Ipswich, MA, USA), N-terminal 7× histidine tag, Factor Xa protease cleavage site, multi cloning site, and *rrnB* gene terminator of the *E. coli* K-12 MG1655 strain were inserted into the Sse8387I-EcoRI site of pHSG298 (Takara Bio Inc.). The pWH1266 *ori* region of the *E. coli-Acinetobacter* shuttle vector pKAMO (25) was amplified using polymerase chain reaction (PCR) and integrated into the resulting plasmid using an In-Fusion HD Cloning Kit (Takara Bio Inc.) to construct the pTAKE vector. The PCR primers and templates used to construct the pTAKE vector are listed in Supplementary Table S1. The pTAKE sequence is available in the Supplementary material (Supplementary Fig. S1).

The pTakeNO plasmid was constructed by inserting the signal peptide of *A. baumannii* Omp38 (KAMO5_06030, signal peptide sequence: KLSRIALATMLVAAPLAAANAGV) into the 5′-end of the target protein-coding sequence of pTAKE. Single-stranded CTAGa-omp38-signalP-A_F (5′-CTAGAATGAAATTGAGTCGTATTGCACTTGCTACTATGCTTGTTGCTGCTCCATTAGCTGCTGCTAATGCTGGCGTAA-3′) and omp38-signalP-ACTAG_R (5′-CTAGTTACGCCAGCATTAGCAGCAGCTAATGGAGCAGCAACAAGCATAGTAGCAAGTGCAATACGACTCAATTTCATT-3′) were annealed to prepare inserts. pTAKE was digested with the restriction enzyme SpeI (Takara Bio Inc.) and ligated using the DNA Ligation Kit, Mighty Mix (Takara Bio Inc.). The pTakeNO plasmid was modified to construct pTakeCO, harboring a C-terminal 7× histidine tag.

The *gfp* and mouse *tnfa* genes were inserted into the pTAKE vector using In-Fusion HD cloning. Genes encoding mNb6-tri-20aa (SARS-Cov-2 nanobody) and ozoralizumab (ATN-103) were codons optimized using Eurofine's GENEius (https://eurofinsgenomics.com/en/resources/other-resources/geneius/), artificially synthesized (Eurofins Genomics, Tokyo, Japan), and then inserted into pTakeCO and pTakeNO, respectively. The PCR primers and templates used to construct the expression plasmids are listed in Supplementary Table S2. The plasmids were transformed into KL037S, ClearColi, and BL21(DE3) cells by electroporation. These bacteria were cultured on LB agar containing 20 µg/mL kanamycin and used as protein-expressing strains.

### Expression and purification of target proteins

Bacteria were cultured for 20 h in LB broth containing 20 µg/mL kanamycin at 37°C with constant shaking (KL037S and ClearColi at 180 rpm; BL21(DE3) at 135 rpm). Overnight bacterial cultures were diluted 10-fold with fresh LB broth and cultivated with constant shaking at 180 rpm until the OD_600_ reached 0.7–1.0. IPTG (FUJIFILM Wako Pure Chemical Industries) was added at a final concentration of 1 mM, and cultivation was continued for an additional 20 h at 37°C with constant shaking at 135 rpm. After centrifugation (13,000*×g* for 15 min at 4°C), the supernatant was collected. The pellets of 2 and 100 mL scale cultures were washed with phosphate-buffered saline, resuspended in 300 µL and 10 mL xTractor Buffer (Takara Bio Inc.), respectively, and incubated for 15 min at 4°C with rotation. After centrifugation (13,000*×g* for 20 min at 4°C), the supernatant was collected as cell lysate. The expressed proteins were purified from the culture supernatant and cell lysates using Ni-NTA agarose (FUJIFILM Wako Pure Chemical Industries).

Purification from the 2-mL culture was conducted using the batch method with 40 µL of Ni-NTA agarose (50% suspension), which was rotated for 1 h at 4°C. Protein-bound Ni-NTA agarose was washed twice with 1 mL of 10 mM imidazole-containing buffer and eluted with 100 µL of 250 mM imidazole-containing buffer. Subsequently, 10 µL of the eluate was analyzed by sodium dodecyl sulfate-polyacrylamide gel electrophoresis (SDS-PAGE) under reducing conditions, and the proteins were visualized by CBB staining.

Purification from 100-mL of culture was conducted using the column method with 1 mL of Ni-NTA agarose (50% suspension). The protein-bound Ni-NTA agarose was washed with 20 mL of 10 mM imidazole-containing buffer and eluted with 1.5 mL of 250 mM imidazole-containing buffer. The buffer composition of the eluate was replaced with pyrogen-free saline, concentrated using Amicon Ultra Centrifugal Filters (Merck Millipore) using 3 KDa filters for GFP and mTNF-α and 30 Kda for mNb6-tri-20aa and ozoralizumab, and then centrifuged at 13,000*×g* for approximately 1 h at 4°C. The protein concentration was quantified using a BCA protein assay kit according to the manufacturer's protocol. Solutions containing 1 µg protein were analyzed by SDS-PAGE under reducing conditions and visualized by CBB staining.

### Measurement of TNF-α activity

The TNF-sensitive mouse fibroblast cell line, L929 (RCB2619), was purchased from RIKEN BRC (Tsukuba, Japan). TNF-α activity was measured according to the method published on the RIKEN BRC website, with slight modifications. The day before the addition of mTNF-α, 1.5 × 10^4^ L929 cells were plated in each well of a 96-well plate. The concentration of purified mTNF-α was measured using a Mouse TNF-alpha DuoSet ELISA (R&D Systems, Minneapolis, MN, USA). The cells were washed with RPMI 1640 medium (FUJIFILM Wako Pure Chemical Industries), followed by three-fold serial dilution of mTNF-α, addition of 4 µg/mL actinomycin D (FUJIFILM Wako Pure Chemical Industries), and incubation for 18 h at 37°C in a 5% CO_2_ atmosphere. The cell survival rate was measured using an MTT assay (Dojindo Laboratories, Kumamoto, Japan).

### Coronavirus neutralization assay using antibodies

The neutralizing activities of the purified recombinant antibodies were measured as previously described (41). The day before viral infection, 10^4^ VeroE6 cells were plated in each well of a 96-well plate. Further, 10-fold serial dilutions of mNb6-tri-20aa (SARS-Cov-2 nanobody) were performed, and ozoralizumab was generated as the negative control antibody in Opti-MEM medium (Thermo Fisher Scientific). Subsequently, the wild-type (Wuhan) or delta strains of coronaviruses were added and incubated for 1 h at 37°C. The virus-antibody solution was then added to the plates cultured with VeroE6 cells at a multiplicity of infection of 0.01. After 3 d of cultivation, an MTT assay was performed, and viral activity was measured based on cell viability.

### Enzyme-linked immunosorbent assay

The Human TNF-alpha DuoSet ELISA was performed according to the manufacturer's protocol, with a slight modification in that the capture antibody was replaced with 4 µg/mL of ozoralizumab (anti human TNF-α nanobody). Furthermore, mNb6-tri-20aa was used as a negative control antibody, whereas human IL-8 (standard in the Human IL-8/CXCL8 DuoSet ELISA) was used as a negative control for cytokines.

### Statistical analysis

Statistical analysis was performed using GraphPad Prism software (GraphPad Software, San Diego, CA, USA). Data are expressed as the mean ± SD. Comparisons between the two groups were performed using an unpaired *t* test. One-way analysis of variance and Tukey's test were used to compare multiple groups. Differences were considered statistically significant at *P*-values of <0.05.

## Supplementary Material

pgae328_Supplementary_Data

## Data Availability

The original datasets are available in a publicly accessible repository. The RNA sequencing data were registered with the DDBJ (BioProject accession no. PRJDB16719).

## Change History

October 3, 2024: The Supplementary Material has been updated to remove author responses to reviewer comments, which had been erroneously included due to a vendor error.
