# Supplementary material for: Development of a novel bacterial production system for recombinant bioactive proteins completely free from endotoxin contamination
Source: PNAS Nexus. 2024 Aug 7;3(8):pgae328. doi: 10.1093/pnasnexus/pgae328 (PMC11331542; doi:10.1093/pnasnexus/pgae328)
Supplement: pgae328_Supplementary_Data [file pgae328_supplementary_data.docx]

*PNAS Nexus*

**Development of a novel bacterial production system for recombinant** **bioactive proteins completely free from endotoxin contamination**

Go Kamoshida^1, 2*^, Daiki Yamaguchi^2^, Yuki Kaya^2^, Toshiki Yamakado^2^, Kenta Yamashita^2^, Moe Aoyagi^2^, Saaya Nagai^2^, Noriteru Yamada^2^, Yu Kawagishi^2^, Mizuki Sugano^1^, Yoshiaki Sakairi^1^, Mikako Ueno^3^, Norihiko Takemoto^4*^, Yuji Morita^1^, Yukihito Ishizaka^3^, Kinnosuke Yahiro^2^

^1^ Department of Infection Control Science, Meiji Pharmaceutical University, Tokyo, Japan

^2^ Laboratory of Microbiology and Infection Control, Kyoto Pharmaceutical University, Kyoto, Japan

^3^ Department of Intractable Diseases, National Center for Global Health and Medicine, Tokyo, Japan

^4^ Pathogenic Microbe Laboratory, Research Institute, National Center for Global Health and Medicine, Tokyo, Japan

* Corresponding authors:

E-mail: kamoshida@my-pharm.ac.jp (GK)

E-mail: ntakemoto@ri.ncgm.go.jp (NT)

**Supporting information**

**Table S1. Primers and templates used to construct the pTAKE vector**

**Table S2. Primers and templates used to construct expression plasmids**

**
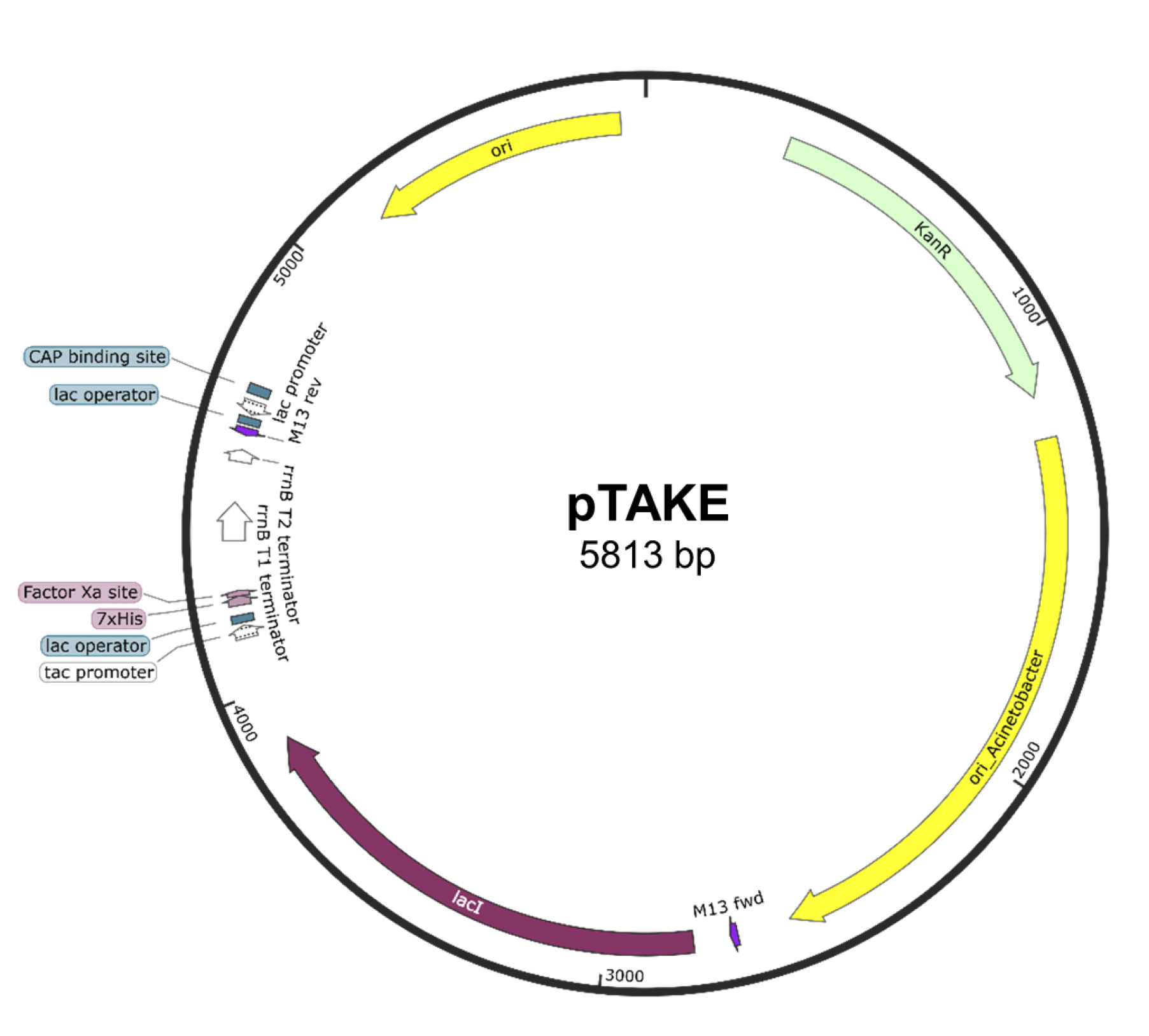
**

**Fig. S1. Vector map of pTAKE-empty**

**Fig. S2. Fluorescence observations of green fluorescent protein (GFP).** Purified GFP from the KL037S and ClearColi strains was diluted to 10 µg/100 µL to make a two-fold dilution series with saline and observed for fluorescence signals. Data are expressed as the means from three independently purified samples.

**Fig. S3. Cytokine expression using an endotoxin-free system.** Purification of mouse interferon (mIFN)-γ (A) and mouse granulocyte colony-stimulating factor (mG-CSF) (B) from the culture supernatants (sup) and cell lysates (lysate) of the 2-mL culture with or without isopropyl-β-D-thiogalactopyranoside (IPTG) induction were conducted using KL037S with pTAKE-mIFN-γ or pTAKE-mGCSF. Purified cytokine fractions were separated using electrophoresis and visualized using Coomassie brilliant blue staining. The arrowhead indicates mIFN-γ.

**
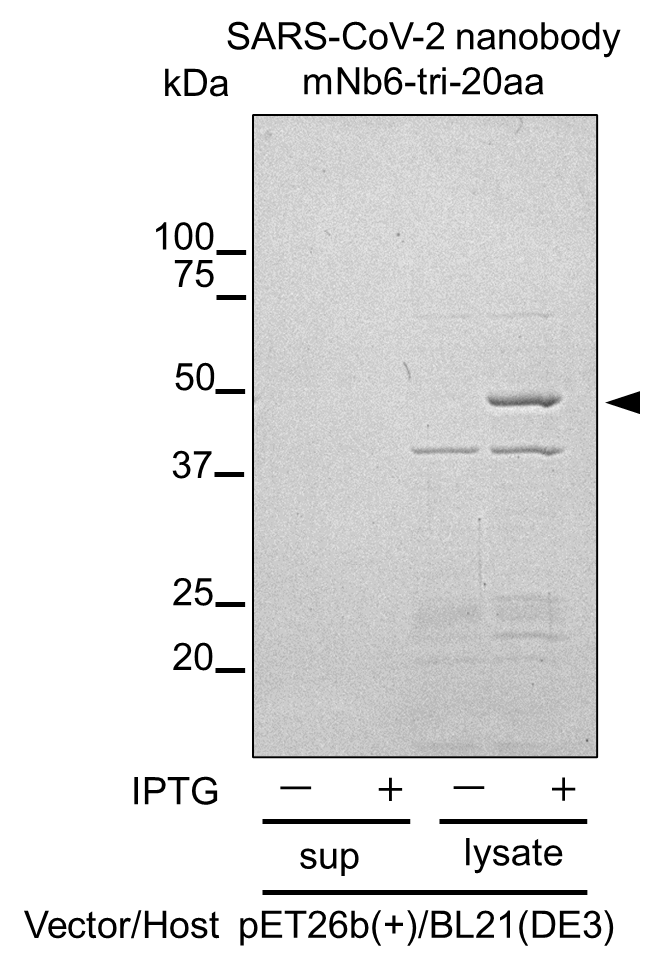
**

**Fig. S4. Existing expression system for mNb6-tri-20aa.** Purified fractions of mNb6-tri-20aa were analyzed using sodium dodecyl sulfate-polyacrylamide gel electrophoresis, followed by visualization with Coomassie brilliant blue staining. Target proteins were expressed in 2-mL cultures with or without isopropyl-β-D-thiogalactopyranoside (IPTG) induction of BL21(DE3) with pET26b(+)-mNb6-tri-20aa and then purified from the culture supernatants (sup) and cell lysates (lysate). Arrowheads indicate mNb6-tri-20aa.

**Fig. S5. Expression and purification of variable domain of heavy chain of heavy chain antibodies by other plasmids.** mNb6-tri-20aa (SARS-CoV-2 nanobody) and ozoralizumab (ATN-103) were purified from the culture supernatants (sup) and cell lysates (lysate) from the 2-mL culture with or without isopropyl-β-D-thiogalactopyranoside (IPTG) induction of KL037S. Experiments were conducted using the KL037S strain harboring pTakeNO-mNb6-tri-20aa (A) or pTakeCO-ozoralizumab (B).

**Fig. S6. Adaptation of Omp38 signal peptide to mouse tumor necrosis factor (mTNF)-α.** Purified fractions of mTNF-α were analyzed using sodium dodecyl sulfate-polyacrylamide gel electrophoresis, followed by visualization with Coomassie brilliant blue staining. Target proteins were expressed in 2-mL cultures with or without isopropyl-β-D-thiogalactopyranoside (IPTG) induction of the KL037S strain with pTakeNO-mTNF-α (A) or pTakeCO-mTNF-α (B) and then purified from the culture supernatants (sup) and cell lysates (lysate). Arrowheads indicate mTNF-α.

**Fig. S7. Expression and purification of antibodies using *Escherichia coli* expression system** (A) Purified fractions of mNb6-tri-20aa (left panel) and ozoralizumab (right panel) were analyzed using sodium dodecyl sulfate-polyacrylamide gel electrophoresis (SDS-PAGE) and visualized using Coomassie brilliant blue (CBB) staining. Target antibodies were expressed in 2-mL cultures with or without isopropyl-β-D-thiogalactopyranoside (IPTG) induction of ClearColi with pET26b(+)-mNb6-tri-20aa or pET26b(+)-ozoralizumab and then purified from the culture supernatants (sup) and cell lysates (lysate). Arrowheads indicate mNb6-tri-20aa (left panel) and ozoralizumab (right panel). (B) mNb6-tri-20aa fractions purified from the lysate of a 100-mL culture of BL21(DE3) with pET26b(+)-mNb6-tri-20aa were separated using SDS-PAGE. CBB staining was performed to visualize whole proteins. The 1-µg (left) or 3-µg (right) purified fractions of mNb6-tri-20aa were analyzed. Arrowheads indicate mNb6-tri-20aa.

**Fig. S8. Growth of the KL037S strain.** ATCC 19606 and KL037S strains with initial OD_600_ 0.001 were grown in Luria–Bertani broth at 37 °C for 24 h. Absorbance at 595 nm was measured hourly. Data are presented as the mean ± the standard deviation; n = 3 per group
